# Influence of oral bacteria on adhesion of Streptococcus mutans and Streptococcus sanguinis to dental materials

**DOI:** 10.1002/cre2.107

**Published:** 2018-06-03

**Authors:** Cindy Nabert‐Georgi, Arne C. Rodloff, Holger Jentsch, Daniel R. Reissmann, Reiner Schaumann, Catalina Suzana Stingu

**Affiliations:** ^1^ Institute for Medical Microbiology and Epidemiology of Infectious Diseases University Hospital of Leipzig Germany; ^2^ Centre for Periodontology, Department of Cariology, Endodontology and Periodontology University Hospital of Leipzig Germany; ^3^ Department of Prosthetic Dentistry, Center for Dental and Oral Medicine University Medical Center Hamburg‐Eppendorf Germany

**Keywords:** adhesion, composite, Streptococcus mutans, Streptococcus sanguinis, zirconia

## Abstract

In this study, the effect of bacterial multispecies communities on the adhesion of *Streptococcus mutans* and *Streptococcus sanguinis* to dental restorative material was investigated. The saliva‐coated specimens of zirconia and composite were incubated with the following combinations: single species, *S. mutans* or *S. sanguinis*; two species, single species combined with other oral streptococci; multiple species, combination of *Actinomyces naeslundii*, *Fusobacterium nucleatum*, and *Prevotella* ssp.; and the two‐species combinations. The adherent bacteria were counted after plating of serial dilutions. Effects of material and bacteria on adhesion of *S. mutans* and *S. sanguinis* were evaluated with multiple linear regression analyses. No significant differences between the materials regarding the adhesion of *S. mutans* and *S. sanguinis* were observed. The adhesion of *S. mutans* was negatively influenced by the presence of other streptococci. Enhancing effects (610.6%) were seen in the presence of *Prevotella intermedia*. The adhesion of *S. sanguinis* decreased in the presence of other bacteria, except *F. nucleatum* (increase of 717.4%). Significant inhibitory effects were detected in the presence of *S. mutans* and *A. naeslundii* (reduction of 95.9% and 78.5%, respectively). The results of this study suggest that adhesion of both types of streptococci to restorative materials is influenced by various bacterial interactions.

## 1 INTRODUCTION

A great variety of bacteria (more than 700 species) can be found in the human oral cavity (Paster, Olsen, Aas, & Dewhirst, 2006). Some of these bacteria are able to colonize surfaces coated by the salivary pellicle. Viridans streptococci followed by *Actinomyces* and a few other genera are able to colonize the tooth surface by binding via adhesins to the proteinaceous pellicle (Kolenbrander et al., 2002). The early colonizers may provide binding sites for other bacteria that cannot directly bind to the pellicle. One of the most important mechanisms of bacterial colonization and dental biofilm formation is coaggregation. Coaggregation only occurs between compatible partner organisms with cell‐surface adhesins on one cell type and cognate receptors on the other (Kolenbrander, Ganeshkumar, Cassels, & Hughes, 1993). *Fusobacterium nucleatum* plays a key role, as it is able to coaggregate with many oral bacteria, early and late colonizers (Kolenbrander, Andersen, & Moore, 1989), and acts as a bridge between them. Interactions between different species of bacteria and between bacteria and the host influence the development of oral biofilms. The formation of dental plaque on tooth surfaces, as well as on restorative materials, may lead to dental caries and periodontal diseases (Axelsson & Lindhe, 1978).

Currently, esthetic tooth‐colored restorations play an important role in dentistry and are preferred by many patients. In the last decade, the role of the silver amalgam has declined (Taut, 2013) and the use of composites has increased. This is mainly due to patient concern about the mercury release of amalgam restorations and partly for esthetic reasons (Burke, Wilson, Cheung, & Mjör, 2001). Composites are widely used as a common filling material in conservative dentistry (Moraschini, Fai, Alto, & Dos Santos, 2015). Current nanohybrid composites are state of the art and can be used as universal composites for anterior and posterior restorations (Mitra, Wu, & Holmes, 2003). They were developed from microhybrid composites by using nanofillers. An increased filler content could be achieved and led to improvements in mechanical properties, as well as excellent esthetics (Mitra et al., 2003), high wear resistance (Turssi, Ferracane, & Serra, 2005), and low polymerization shrinkage (Ferracane, 1995).

This trend is also reflected in the metal‐free restorations of prosthodontics. Besides feldspar and lithium disilicate ceramics, high‐strength zirconium oxides are specifically used as framework for crowns and bridges and offer an alternative and esthetic advantage to the typical metal ceramic systems. Since the advent of full‐contour restorations, their field of application increased as well as the surface area in direct contact with the oral cavity. The material used most among the zirconium oxides is yttrium‐stabilized zirconia ceramic (yttria‐stabilized tetragonal zirconia polycrystal; Denry & Kelly, 2008).

Many studies focus only on the adhesion of a single streptococcal species to restorative materials (Poggio et al., 2009; Rosentritt et al., 2008; Rosentritt, Behr, Burgers, Feilzer, & Hahnel, 2009; Takatsuka et al., 2000; Zalkind, Keisar, Ever‐Hadani, Grinberg, & Sela, 1998). To our knowledge, no studies have analyzed multiple‐species adhesion on restorative materials or the influence of other bacteria on the adhesion of oral streptococci on such materials. With this study, the effect of bacterial multispecies associations on the adhesion of *Streptococcus mutans*, as a representative of cariogenic streptococci (Loesche, 1986), and *Streptococcus sanguinis*, as a presumably commensal early colonizer (Nyvad & Kilian, 1987), on dental restorative materials was investigated.

## 2 MATERIAL AND METHODS

### 2.1 Specimens

Rectangular specimens with an identical surface of 227 mm^2^ of composite (6.9 × 13.0 × 1.2 mm; IPS Empress® Direct Enamel, Ivoclar Vivadent, Schaan, Liechtenstein) and ceramic (8.1 × 12.5 × 0.6 mm; In‐Ceram® YZ for inLab®, VITA Zahnfabrik, Bad Säckingen, Germany) were used in this study.

The composite specimens were prepared as follows: A block of composite was prepared by layers of maximum 2 mm thickness; each increment was light cured for 40 s (HS‐LED 1200, Henry Schein, Langen, Germany). Subsequently, using a saw microtome (Leitz 1600, Ernst Leitz Wetzlar GmbH, Wetzlar, Germany), the block was cut from all sides and identical disks were made. Afterwards, the disks were polished with silicone polisher with diamond crystallites for 20 s on each side (Optrapol® Next Generation, Ivoclar Vivadent, Schaan, Liechtenstein) under cool water using a slow‐speed hand piece rotating at 10,000 rpm.

The ceramic specimens were prepared as follows: The presintered zirconia block was cut in identical disks and sintered afterwards. The disks were then polished 20 s to high gloss with a two‐step diamond polishing system for zirconia by a hand piece rotating at 10,000 rpm (Diacera, EVE Ernst Vetter GmbH, Pforzheim, Germany). The polishing procedure was carried out using commercially available polishers in order to create a surface similar with the one used in clinical practice.

### 2.2 Saliva coating of specimens

Artificial saliva was prepared as described by Rosentritt et al. (2009). Therefore, albumin (40 μg/ml; Albumin, bovine; Sigma‐Aldrich, St. Louis, USA), amylase (1 mg/ml; α‐Amylase from hog pancreas; Fluka Biochemika, Buchs, CH), lysozyme (10 μg/ml; Lysozyme from hen egg white; Fluka Biochemika, Buchs, CH), and mucin (850 mg/L; Mucin from porcine stomach; Sigma‐Aldrich, St. Louis, USA) were added to phosphate buffered saline (PBS, Sigma‐Aldrich, St. Louis, USA). Before use, the saliva was stored at −20 °C. Six specimens that were decontaminated with ethanol 90% vol. for 10 min were transferred to a six‐well cell plate (Nunclon™ Delta Surface, Nunc A/S, Roskilde, Denmark). Each well was filled with 1.5‐ml artificial saliva and incubated for 1 hr at 37 °C on a thermo‐shaking device (G24 Environmental Incubator Shaker, New Brunswick Scientific Co. Inc., Enfield, USA).

### 2.3 Species combinations and culture conditions

The following strains were used in this study: Streptococcus anginosus DSMZ 20563, *Streptococcus gordonii* DSMZ 6777, *Streptococcus mitis* DSMZ 12643, *Streptococcus oralis* DSMZ 20627, *S. mutans* DSMZ 20523, *S. sanguinis* DSMZ 20567, *Actinomyces naeslundii* ATCC 12104, *F. nucleatum* ATCC 25586, *Prevotella intermedia* DSMZ 20706, and *Prevotella nigrescens* DSMZ 13386. Streptococci and anaerobic strains (including actinomyces) were cultured on Columbia agar for 48 hr at 37 °C in a CO_2_‐enriched atmosphere, respectively, 7 days at 37 °C in an anaerobic atmosphere (anaerobic gas mixture: 15% CO_2_, 5% H_2_O_2_, and 80% NO_2_; Whitley MG1000 Anaerobic Workstation, Don Whitley Scientific, UK). The cells were harvested from the plates with plastic loops and suspended separately in tubes with brain heart infusion broth to a final cell density of 0.5 McFarland (Densimat, bioMérieux, Lyon, France).

### 2.4 Adhesion tests

Two milliliters of each bacterial suspension (Table 1) were added to the saliva‐coated specimens in the six‐well plate (Table 1). The plate was placed on a shaker for 24 hr at 37 °C. After incubation, specimens were rinsed gently 3 times in 30‐ml PBS to remove loosely adhered bacteria. The attached cells were then dispersed by vortex in 2‐ml PBS for 2 min. Tenfold serial dilutions of the adherent cell suspension and the remaining bacteria in the six‐well plate were prepared, and 0.1 ml of each dilution was plated onto Columbia agar plates and were incubated at 37 °C for 48 hr in CO_2_‐enriched atmosphere for Combinations 1 and 2 and at 37 °C for 7 days in an anaerobic workstation for Combinations 3–5. Control plates containing one single species were performed for each experiment. Based on their morphology, all the colonies were counted and visually identified (Kaiser prolite basic; Kaiser Fototechnik GmbH & Co. KG, Buchen, Germany). The remaining bacterial suspensions from all six wells were used as a control for growth. In case of doubt, we further isolated the colonies and identified them biochemically using Rapid ID 32 Strep system (bioMérieux, Lyon, France). For each experiment, the results were expressed in colony‐forming unit (CFU)/ml and converted into CFU/mm^2^ (adherent cells on the specimens) using Equations (1) and (2). All the experiments were carried out twice.

(1) $$\mathrm{CFU}/\mathrm{ml}=n\;\cdot \;\frac{1}{\text{Dilution}\;\left(10^{-1}-10^{-6}\right)}\;\cdot \;\frac{1}{\text{Volume}\;\left(100\;\mathrm{\mu l}\right)},$$

(2) $$\mathrm{CFU}/{\mathrm{mm}}^{2}=\frac{\frac{\mathrm{CFU}}{\mathrm{ml}}\;\cdot \;2\;\mathrm{ml}}{227\;{\mathrm{mm}}^{2}}\;\left(\text{surface size of composite and ceramic}\right).$$

**Table 1:** Bacterial combinations

One‐species suspensions—*S. mutans* or *S. sanguinis* Two‐species suspensions—*S. mutans* or *S. sanguinis* combined with S. anginosus, S. mitis, *S. oralis*, or *S. gordonii* Three‐species suspensions—Combination from *A. naeslundii* and the two‐species combinations Four‐species suspensions—Combination from *F. nucleatum* and the three‐species combinations Five‐species suspensions—Combination from P. intermedia, respectively P. nigrescens with the four‐species combinations

*Note*. *S. mutans* = *Streptococcus mutans*; *S. sanguinis* = *Streptococcus sanguinis*; S. anginosus = Streptococcus anginosus; S. mitis = *Streptococcus mitis*; *S. oralis* = *Streptococcus oralis*; *S. gordonii* = *Streptococcus gordonii*; *A. naeslundii* = *Actinomyces naeslundii*; *F. nucleatum* = *Fusobacterium nucleatum*; P. intermedia = *Prevotella* intermedia; P. nigrescens = *Prevotella nigrescens* .

### 2.5 Data analysis

Effects of material and the presence of specific bacteria (predictors) on adhesion of *S. mutans* and *S. sanguinis* were computed using multiple linear regression analyses. Due to nonnormality, the natural logarithm of the CFU values of *S. mutans* and *S. sanguinis* from 52 assessments (different combinations of predictors) for each case were used as criterion variables. The resulting regression coefficients for the respective material and bacteria in the regression models were subsequently delogarithmized and converted to percentages, representing the mean change of adhesion of *S. mutans* and *S. sanguinis* caused by the corresponding material and bacteria, respectively. However, it has to be noted that effects are not additive like in regular linear regression analyses but multiplicative due to delogarithmizing of the coefficients derived from the regression models.

All analyses were performed using the statistical software package STATA MP 13.1 (Stata Statistical Software. StataCorp LP, College Station, TX, USA), with the probability of a Type I error set at 0.05.

## 3 RESULTS

Both restorative materials showed no statistically significant effect on the adhesion of *S. mutans* and *S. sanguinis* (Tables 2 and 3).

**Table 2:** Impact of material and bacteria on adherence of *S. mutans*; results from multiple linear regression analysis, coefficients after delogarithmizing representing the multiplicative effect of each predictor (independent variable) on adherence of *S. mutans* as the criterion (dependent variable), after statistically controlling for the impact of the other predictors, that is, they were hold constant

Criterion	Predictor	Coef.	95% CI	Change in %	*p* value	
*S. mutans*						
	Material (zirconia/composite)	2.01	[0.63, 6.38]	+100.7	.230	
	*S. anginosus*	0.01	[0.00, 0.41]	−98.7	.015	
	*S. gordonii*	0.07	[0.00, 2.16]	−93.0	.125	
	*S. mitis*	0.11	[0.00, 3.38]	−89.1	.200	
	*S. oralis*	0.16	[0.00, 4.82]	−84.5	.280	
	*S. sanguinis*	0.09	[0.00, 2.88]	−90.7	.170	
	*A. naeslundii*	3.53	[0.55, 22.76]	+253.1	.179	
	*F. nucleatum*	1.70	[0.26, 10.98]	+70.3	.567	
	*P. intermedia*	7.11	[1.10, 45.81]	+610.6	.040	
	*P. nigrescens*	0.62	[0.10, 4.01]	−37.7	.611	

*Note*. *S. mutans* = *Streptococcus mutans*; *S. sanguinis* = *Streptococcus sanguinis*; S. anginosus = Streptococcus anginosus; S. mitis = *Streptococcus mitis*; *S. oralis* = *Streptococcus oralis*; *S. gordonii* = *Streptococcus gordonii*; *A. naeslundii* = *Actinomyces naeslundii*; *F. nucleatum* = *Fusobacterium nucleatum*; P. intermedia = *Prevotella* intermedia; P. nigrescens = *Prevotella nigrescens* .

**Table 3:** Impact of material and bacteria on adherence of *S. sanguinis*; results from multiple linear regression analysis, coefficients after delogarithmizing representing the multiplicative effect each predictor (independent variable) on adherence of *S. sanguinis* as the criterion (dependent variable), after statistically controlling for the impact of the other predictors, that is, they were hold constant

Criterion	Predictor	Coef.	95% CI	Change in %	*p* value	
*S. sanguinis*						
	Material (zirconia/composite)	0.68	[0.33, 1.41]	−32.4	.286	
	*S. anginosus*	0.59	[0.07, 5.19]	−41.0	.626	
	*S. gordonii*	0.48	[0.05, 4.23]	−51.9	.501	
	*S. mitis*	0.49	[0.06, 4.27]	−51.5	.505	
	*S. mutans*	0.04	[0.00, 0.36]	−95.9	.005	
	*S. oralis*	0.44	[0.05, 3.87]	−56.1	.449	
	*A. naeslundii*	0.21	[0.07, 0.70]	−78.5	.012	
	*F. nucleatum*	8.17	[2.51, 26.59]	+717.4	.001	
	*P. intermedia*	0.63	[0.19, 2.04]	−37.2	.430	
	*P. nigrescens*	0.44	[0.13, 1.42]	−56.4	.163	

*Note*. *S. mutans* = *Streptococcus mutans*; *S. sanguinis* = *Streptococcus sanguinis*; S. anginosus = Streptococcus anginosus; S. mitis = *Streptococcus mitis*; *S. oralis* = *Streptococcus oralis*; *S. gordonii* = *Streptococcus gordonii*; *A. naeslundii* = *Actinomyces naeslundii*; *F. nucleatum* = *Fusobacterium nucleatum*; P. intermedia = *Prevotella* intermedia; P. nigrescens = *Prevotella nigrescens* .

### 3.1 Effects on adhesion of *S. mutans*

The adhesion of *S. mutans* was negatively influenced in combination with other streptococci and P. nigrescens. Enhancing effects of 610.6%, approximately a sevenfold increase (*p* = .040), were observed in the presence of P. intermedia. In addition, *A. naeslundii* and *F. nucleatum* promoted the adhesion of *S. mutans*; however, unlike P. intermedia, these were not significant (Table 2).

### 3.2 Effects on adhesion of *S. sanguinis*

The adhesion of *S. sanguinis* decreased in the presence of all other bacteria tested, except of *F. nucleatum*. In the presence of *F. nucleatum*, the adhesion was significantly increased by 717.4% (more than eightfold; *p* = .001). Significant inhibitory effects were detected in the presence of *S. mutans* and *A. naeslundii* with a reduction of 95.9% (*p* = .005) and 78.5% (*p* = .012), respectively (Table 3).

## 4 DISCUSSION

There are many studies that focus on bacterial adhesion to dental materials (Poggio et al., 2009; Rosentritt et al., 2008, 2009; Takatsuka et al., 2000; Zalkind et al., 1998). The appearance of new materials combined with new scientific knowledge about oral biofilm creates a need for further research.

It is well known that several factors can influence the adhesion of bacteria to different dental materials. One of the most important factor influencing bacterial adhesion is the salivary pellicle (Takatsuka et al., 2000). Saliva coating leads to a homogenization of contact angles and a more hydrophilic surface (Sardin, Morrier, Benay, & Barsotti, 2004; Takatsuka et al., 2000). To mimic the conditions in the oral cavity, each experiment was performed with saliva coating. Artificial saliva was chosen based on the protocol of Rosentritt et al. (2009) to minimize interindividual variation. However, because of different and complex protein adsorption patterns on various substrata and the broad range of proteins in human saliva that interact with oral bacteria specifically, it might be possible that in vivo adhesion patterns of bacteria differ from this in vitro results.

The experimental setup affects bacterial adhesion, particularly regarding variable salivary flow rates and shear stress (Mohamed, Rainier, & Ross, 2000). This study was set up as a semistatic model created by the thermo‐shaking device that was partially able to simulate shear stress and was similar to the semistatic conditions in other in vitro studies (Rosentritt et al., 2009; Sardin et al., 2004; Takatsuka et al., 2000). In addition, culture medium, culture conditions, or growth phase of the bacteria may influence early bacterial colonization in vitro (Sardin et al., 2004). To preclude different bacterial adhesion resulting from different growth conditions, all strains were prepared identically.

Previous in vitro studies focused mainly on the adhesion of a single streptococcal species to dental material surfaces (Poggio et al., 2009; Rosentritt et al., 2008, 2009; Takatsuka et al., 2000). Although there are more than 700 species in the oral cavity, no emphasis was made on bacterial interactions. The aim of this study was to investigate the effects of bacterial multispecies associations on the adhesion of *S. mutans* and *S. sanguinis* to two dental materials, composite and zirconia.

The results showed no statistically significant differences between materials regarding the adhesion of *S. mutans* and *S. sanguinis*. This is in contrast to previous studies that reported a greater level of adhesion of *S. mutans* (Zalkind et al., 1998) on composites than on other materials, such as ceramics or amalgam. The reason was assumed to be a degeneration of material after a longer period (up to 35 days; Willershausen, Callaway, Ernst, & Stender, 1999) or that special monomers or fillers may promote bacterial adhesion. However, Rosentritt et al. (2008) observed great differences between veneering composites for crowns and bridges. They found a significantly higher adhesion of *S. mutans* to the composite samples compared with a range of ceramics; however, there were also a few composites with adhesion comparable with that of the ceramic samples. The later findings are in accordance with our research. *S. mutans* and *S. sanguinis* adhesion did not differ significantly between composite and zirconia. Poggio et al. (2009) revealed less adhesion of *S. mutans* to some of the different types of direct filling composites that were tested. One of these composites with less adhesion was a nanohybrid composite. The IPS Empress® Direct Enamel used in this study, which is a nanohybrid composite as well, probably has similar low adhesion properties. In terms of ceramics, several previous in vitro (Rosentritt et al., 2008, 2009) and in vivo (Hahn, Weiger, Netuschil, & Brüch, 1993) studies reported a low or intermediate streptococcal colonization of ceramic samples. Only a few minor differences were reported with streptococcal adhesion to various ceramics and YZ zirconia in single species experiments.

Early colonizers, such as *S. sanguinis*, *S. oralis*, S. mitis, and *Actinomyces* spp., colonize the acquired pellicle during the initial phase of biofilm formation (Marsh & Bradshaw, 1995). They bind irreversibly to the pellicle by means of adhesins and fimbriae and with the help of stereochemical interactions (Marsh & Bradshaw, 1995). Extracellular polysaccharides play an important role in the adhesion of oral bacteria to surfaces. Mutans streptococci produce glycans facilitating adhesion of the initial colonizers (Vickerman & Jones, 1995). In this study, the adhesion of *S. mutans* was negatively influenced by the presence of other streptococci. The slightly negative influences of the other streptococci on *S. mutans* can be explained as an inverse relation to caries development by the presence of the members of the Sanguinis group of oral streptococci (Loesche, Rowan, Straffon, & Loos, 1975). It is suggested that the production of hydrogen peroxide by the members of the Sanguinis group is partially responsible by antagonizing the growth of *S. mutans* (Kreth, Zhang, & Herzberg, 2008). *S. gordonii* is known to produce challisin, which is a protease that degrades the competence‐stimulating peptide signal from *S. mutans* (Wang, Deutch, Hong, & Kuramitsu, 2011; Wang & Kuramitsu, 2005). The ability to inhibit mutacin production by degrading competence‐stimulating peptide of *S. mutans* was also demonstrated for *S. sanguinis*, S. mitis, and *S. oralis* (Wang & Kuramitsu, 2005), but no specific protease could be identified. Wang et al. (2011) revealed that the protease expression of Sanguinis organisms can alter the potential of *S. mutans*. Thus, different members of this group can produce differential effects on *S. mutans* virulence gene expression (Wen, Yates, Ahn, & Burne, 2010). All these mechanisms explain the negative influence of the Sanguinis group on the adhesion of *S. mutans* on both materials. Our results also revealed enhancing effects of *S. mutans* adhesion in the presence of *A. naeslundii*, *F. nucleatum*, and P. intermedia. This effect was only statistically significant (increase of 610.6%) for P. intermedia. Interestingly, the adhesion of *S. mutans* was inhibited by P. nigrescens (reduction of 37.7%) but enhanced in the presence of P. intermedia. We cannot make any comments about the influence of S. anginosus on the adhesion of *S. mutans* on both materials. Both two species experiments (repeatedly twice) showed no attaching bacteria on both materials but also no or little growth of these bacteria in the remaining suspensions.

The found adhesion of *S. sanguinis* decreased in the presence of other bacteria, except *F. nucleatum*, and significant inhibitory effects were seen in the presence of *S. mutans* and *A. naeslundii* (reduction of 95.9% and 78.5%, respectively). The decrease of *S. sanguinis* adhesion in the presence of *S. mutans* may be the result of competitive effects of early colonizers and attributed to the similar metabolic activities or adhesion binding sites on saliva coated surfaces (Nobbs, Zhang, Khammanivong, & Herzberg, 2007). The presence of oxygen seems to play a great role in interspecies competition. Streptococcal hydrogen peroxide is an important competitive factor and sensing molecule between oral bacteria. The hydrogen peroxide is produced by pyruvate oxidase, as a by‐product of aerobic metabolism in *S. sanguinis* (Carlsson, Edlund, & Lundmark, 1987). Kreth et al. (2008) demonstrated pyruvate oxidase‐dependent inhibition of *S. mutans* growth under aerobic conditions. Conversely, *S. mutans* was able to inhibit *S. sanguinis* during anaerobic growth. A decreased oxygen content can also be found in deep periodontal pockets of periodontitis patients. Stingu, Eschrich, Rodloff, Schaumann, and Jentsch (2008) found a correlation between aggressive periodontitis and the loss of peroxidogenic *S. sanguinis*. It is possible that *A. naeslundii* also benefits from less hydrogen peroxide and competes with *S. sanguinis* as an early colonizer. The anaerobic conditions may promote the adhesion of the facultative anaerobic *A. naeslundii* and creates conditions for the maturation of the biofilm. *A. naeslundii*, together with streptococci, is a significant early colonizer of the tooth surface. It has two major fimbriae. Type I binds to the salivary pellicle, and Type II is involved in interbacterial binding (Yeung, 1999). Furthermore, *F. nucleatum* was the only bacteria that significantly increased (717.4%) the adhesion of *S. sanguinis* to both tested materials. It is well known that *F. nucleatum*, an obligate anaerobic, is the most important coaggregating partner for almost all other bacteria realizing a bridge between the early and late colonizers (Kolenbrander et al., 1989, 2002). The coaggregation between *F. nucleatum* and *S. sanguinis* mediated by a surface protein can explain the increased adhesion of *S. sanguinis* (Kaplan, Lux, Haake, & Shi, 2009).

Oral streptococci are growing well in both CO_2_‐enriched atmosphere and anaerobic workstation. All experiments involving anaerobic bacteria were performed from the beginning in the anaerobic workstation. The adhesion of *S. sanguinis* increased in the presence of *F. nucleatum* but not in the presence of P. intermedia or P. nigrescens. So we have to assume that it was not the anaerobic atmosphere but bacteria that influenced it.

In conclusion, within the limits of this study, it is suggested that adhesion of both *S. mutans* and *S. sanguinis* to zirconia or composite is influenced by various multiple bacterial interactions. Further in vitro studies are needed to monitor chemical changes on the material surfaces in the presence of bacteria and the metabolic activity of the adhered streptococci under aerobic and anaerobic conditions. Possible metabolic interactions between species and their capacity to survive after longer periods of time (48 hr) should be monitored.

## Supporting information

Data S1 Supporting information item
