# Supplementary material for: Influence of oral bacteria on adhesion of Streptococcus mutans and Streptococcus sanguinis to dental materials
Source: Clin Exp Dent Res. 2018 Jun 3;4(3):72–7. doi: 10.1002/cre2.107 (PMC6010874; doi:10.1002/cre2.107)
Supplement: Supplementary file 1 [file CRE2-4-72-s001.docx]

S. mutans		Zirconia: attached cells in CFU/mm^2	Zirconia: attached cells in CFU ml^{-1}	Zirconia: remaining bacterial suspension in CFU ml^{-1}	Composite: attached cells in CFU/mm^2	Composite: attached cells in CFU ml^{-1}	Composite: remaining bacterial suspension in CFU ml^{-1}
single	SM	**37812**	4291667	3400000000	**58062**	6590000	3608000000
two species	SM+SA	**0**	0	0	**0**	0	2000
	SM+Smi	**16784**	1905000	3030000000	**55947**	6350000	2870000000
	SM+S0	**18722**	2125000	3000000000	**295154**	33500000	2900000000
	SM+SG	**18855**	2140000	770000000	**5286**	600000	1190000000
	SM+SS	**4361**	495000	629000000	**47283**	5366667	910000000
three species	SM+SA+AN	**3877**	440000	1380000000	**4846**	550000	3030000000
	SM+Smi+AN	**17621**	2000000	2440000000	**74009**	8400000	6200000000
	SM+S0+AN	**45521**	5166667	3000000000	**15815**	1795000	7750000000
	SM+SG+AN	**4405**	500000	1660000000	**38326**	4350000	2390000000
	SM+SS+AN	**2643**	300000	1170000000	**4082**	463333	2160000000
four species	SM+SA+AN+FN	**124229**	14100000	1000000000	**22555**	2560000	1190000000
	SM+Smi+AN+FN	**8855**	1005000	2430000000	**18297**	2076667	2900000000
	SM+S0+AN+FN	**18502**	2100000	1520000000	**23348**	2650000	2800000000
	SM+SG+AN+FN	**3965**	450000	1640000000	**8634**	980000	2480000000
	SM+SS+AN+FN	**15742**	1786667	1190000000	**62467**	7090000	700000000
five species A	SM+SA+AN+FN+PI	**91630**	10400000	1182000000	**227313**	25800000	2470000000
	SM+Smi+AN+FN+PI	**15683**	1780000	1940000000	**320705**	36400000	2290000000
	SM+S0+AN+FN+PI	**33979**	3856667	1470000000	**667841**	75800000	3480000000
	SM+SG+AN+FN+PI	**55947**	6350000	2000000000	**569163**	64600000	2520000000
	SM+SS+AN+FN+PI	**45551**	5170000	1660000000	**629075**	71400000	1270000000
five species B	SM+SA+AN+FN+PN	**20881**	2370000	1420000000	**4361**	495000	1210000000
	SM+Smi+AN+FN+PN	**14405**	1635000	2280000000	**9515**	1080000	2550000000
	SM+S0+AN+FN+PN	**21968**	2493333	1940000000	**5551**	630000	1820000000
	SM+SG+AN+FN+PN	**12907**	1465000	3560000000	**6696**	760000	1450000000
	SM+SS+AN+FN+PN	**19251**	2185000	855000000	**22863**	2595000	643000000

Abbreviations: SM= *S. mutans*, SS= *S.sanguinis*, Smi= *S. mitis*, SO= *S. oralis*, SG= *S.gordonii*, AN=*A.naeslundii*, FN= *F. nucleatum*, PI= *Prevotella intermedia*, PN= *P. nigrescens*

S. sanguinis		Zirconia: attached cells in CFU/mm^2	Zirconia: attached cells in CFU ml^{-1}	Zirconia: remaining bacterial suspension in CFU ml^{-1}	Composite: attached cells in CFU/mm^2	Composite: attached cells in CFU ml^{-1}	Composite: remaining bacterial suspension in CFU ml^{-1}
single	SS	**79883**	9066667	1946000000	**43759**	4966667	3560000000
two species	SS+SA	**63084**	7160000	670000000	**8943**	1015000	1175000000
	SS+Smi	**13392**	1520000	1950000000	**26432**	3000000	1850000000
	SS+S0	**2044**	232000	1910000000	**3731**	423500	1770000000
	SS+SG	**86784**	9850000	1140000000	**14097**	1600000	1790000000
	SS+SM	**89912**	10205000	1126000000	**19559**	2220000	1380000000
three species	SS+SA+AN	**22761**	2583333	800000000	**3154**	358000	1510000000
	SS+SMi+AN	**13128**	1490000	1620000000	**3634**	412500	1990000000
	SS+S0+AN	**23436**	2660000	1640000000	**2026**	230000	1250000000
	SS+SG+AN	**12482**	1416667	850000000	**3062**	347500	1730000000
	SS+SM+AN	**1762**	200000	360000000	**62**	7000	70000000
four species	SS+SA+AN+FN	**83700**	9500000	1050000000	**174890**	19850000	1030000000
	SS+SMi+AN+FN	**121586**	13800000	1500000000	**59031**	6700000	1800000000
	SS+S0+AN+FN	**116740**	13250000	1450000000	**101322**	11500000	1360000000
	SS+SG+AN+FN	**29956**	3400000	1480000000	**27753**	3150000	1540000000
	SS+SM+AN+FN	**837**	95000	539500000	**1057**	120000	337500000
five species A	SS+SA+AN+FN+PI	**15639**	1775000	1050000000	**81057**	9200000	770000000
	SS+SMi+AN+FN+PI	**27753**	3150000	1790000000	**26432**	3000000	1810000000
	SS+S0+AN+FN+PI	**21145**	2400000	1540000000	**82379**	9350000	920000000
	SS+SG+AN+FN+PI	**6828**	775000	1620000000	**41850**	4750000	1710000000
	SS+SM+AN+FN+PI	**2070**	235000	443000000	**9104**	1033333	631500000
five species B	SS+SA+AN+FN+PN	**62115**	7050000	820000000	**5771**	655000	1340000000
	SS+SMi+AN+FN+PN	**21498**	2440000	1297000000	**19971**	2266667	1800000000
	SS+S0+AN+FN+PN	**17621**	2000000	1580000000	**107930**	12250000	1280000000
	SS+SG+AN+FN+PN	**69163**	7850000	1660000000	**45815**	5200000	1650000000
	SS+SM+AN+FN+PN	**586**	66500	215000000	**417**	47333	238500000

Abbreviations: SM= *S. mutans*, SS= *S.sanguinis*, Smi= *S. mitis*, SO= *S. oralis*, SG= *S.gordonii*, AN=*A.naeslundii*, FN= *F. nucleatum*, PI= *Prevotella intermedia*, PN= *P. nigrescens*
